# Protective Effect of Edaravone in Primary Cerebellar Granule Neurons against Iodoacetic Acid-Induced Cell Injury

**DOI:** 10.1155/2015/606981

**Published:** 2015-10-18

**Authors:** Xinhua Zhou, Longjun Zhu, Liang Wang, Baojian Guo, Gaoxiao Zhang, Yewei Sun, Zaijun Zhang, Simon Ming-Yuen Lee, Pei Yu, Yuqiang Wang

**Affiliations:** ^1^Institute of New Drug Research and Guangdong Province Key Laboratory of Pharmacodynamic Constituents of Traditional Chinese Medicine, Jinan University College of Pharmacy, Guangzhou 510632, China; ^2^State Key Laboratory of Quality Research in Chinese Medicine and Institute of Chinese Medical Sciences, University of Macau, Avenue Padre Tomás Pereira S.J., Macau

## Abstract

Edaravone (EDA) is clinically used for treatment of acute ischemic stroke in Japan and China due to its potent free radical-scavenging effect. However, it has yet to be determined whether EDA can attenuate iodoacetic acid- (IAA-) induced neuronal death *in vitro*. In the present study, we investigated the effect of EDA on damage of IAA-induced primary cerebellar granule neurons (CGNs) and its possible underlying mechanisms. We found that EDA attenuated IAA-induced cell injury in CGNs. Moreover, EDA significantly reduced intracellular reactive oxidative stress production, loss of mitochondrial membrane potential, and caspase 3 activity induced by IAA. Taken together, EDA protected CGNs against IAA-induced neuronal damage, which may be attributed to its antiapoptotic and antioxidative activities.

## 1. Introduction

Stroke is the second leading cause of mortality among most developed countries [1]. Cerebral ischemia is the most common type of stroke and accounts for 87% of all stroke cases [2]. Brain hypoxia and glucose deprivation are the primary pathophysiological features of cerebral ischemia, which lead to cerebral infraction [3]. After hypoxia, the balance between generation and clearance of reactive oxidative species (ROS) is compromised, and overproduction of ROS as byproducts by mitochondria may result in activation of mitochondria-dependent apoptotic pathway in acute ischemia stroke [4]. More recent lines of evidence have suggested that an excessive ROS generation may lead to mitochondrial membrane depolarization to induce the apoptosis cascade, which results in functional and structural damage to neuronal cells [5]. Mitochondrial dysfunction and excessive oxidative stress play a vital role in the pathogenesis of neurodegenerative diseases, including cerebral ischemia [3, 6]. Therefore, neuroprotective agents that scavenge free radicals and maintain mitochondrial function should be an effective therapeutic strategy for treating ROS-related disorders, especially ischemic stroke [7].

Edaravone (EDA) has been used to treat acute cerebral infraction since 2001, which is the first novel free radical scavenger approved in Japan [8, 9]. EDA has been shown to quench hydroxyl, peroxyl, and superoxide radicals and inhibit free radical-mediated lipid peroxidative damage in a rat middle cerebral artery occlusion (MCAO) model [10]. Furthermore, recent mechanistic research found that EDA suppressed delayed neuronal death induced by reperfusion, reduced long-term inflammatory reaction, and inhibited the expression of vascular endothelial growth factor mediated by the ischemic cascade [8, 11]. Thus, the therapeutic benefit of EDA is attributed not only to its antioxidative activity but also to its ability to regulate various signaling pathways.

Several studies have shown that iodoacetic acid (IAA) treatment could closely mimic the hypoxic/ischemia condition in nerve cells* in vitro *[12–14]. IAA, a chemical ischemia stimulus, inhibits the glycolytic enzyme glyceraldehyde 3-phosphate dehydrogenase irreversibly and induces cell death accompanied with an increase in ROS production, mitochondrial dysfunction, and loss of ATP [15–17]. These responses are very similar to changes observed in animal models of ischemic stroke [18]. Cerebellar granular neurons (CGNs) are primary rat neurons, obtained from cerebellum, which contain the largest homogeneous neuronal population in the brain, which have been widely used as an* in vitro *model for investigating the cellular and molecular mechanisms underlying neuronal apoptosis in neurodegenerative disorders [19–21]. Previously, researchers have demonstrated that IAA reduced cell viability via increasing ROS production in CGNs [22, 23]. It has yet to be determined whether EDA can attenuate IAA-induced neuronal death* in vitro*. Therefore, we herein used the* in vitro* CGNs model exposed to IAA to study the neuronal protective effects of EDA and to explore the possibility of its underlying mechanisms of action.

## 2. Materials and Methods

### 2.1. Chemicals and Reagents

EDA was purchased from Aladdin Reagent Co. (Shanghai, China). MTT, fluorescein diacetate (FDA), propidium iodide (PI), 2′,7′-dichlorofluorescein diacetate (DCFH-DA), DNase, poly-L-lysine, and cytosine-D-arabinofuranoside were obtained from Sigma-Aldrich (St. Louis, MO, USA). Basal medium Eagle (BME), fetal bovine serum, penicillin, and streptomycin were purchased from Invitrogen (Carlsbad, CA, USA). Mitochondrial membrane potential assay kit and caspase 3 activity assay kit were obtained from Beyotime (Shanghai, China). The cytotoxicity detection kit (LDH) was purchased from Roche Diagnostics (Mannheim, Germany).

### 2.2. Primary Cultures of Cerebellar Granule Neurons and Drug Treatment

CGNs were isolated from postnatal 8-day-old Sprague-Dawley rat pups (15–20 g) from the Animal Care Facility of Sun Yat-sen University according to the protocol described by Bilimoria and Bonni [24]. The density of viable cells in suspension was determined by cell count using trypan blue and adjusted to 2 × 10^6^ cells/mL. Neurons were seeded into 96-well pates or 12-well plates which were precoated with 50 *μ*g/mL poly-L-lysine and maintained in a humidified incubator with 5% CO_2_ in air at 37°C. Twenty-four hours after plating, cytosine-D-arabinofuranoside (final concentration: 10 *μ*M) was added to the culture medium to arrest the proliferating of nonneuronal cells. All animal studies were conducted according to guidelines of the Experimental Animal Care and Use Committee of Jinan University. The experimental protocols were approved by the Ethics Committee for Animal Experiments of Jinan University.

Unless otherwise stated, on day 7 of* in vitro* culture, CGNs were pretreated with EDA (3, 10, and 30 *μ*M) for 2 h. IAA (50 *μ*M) was then added for incubation for another 4 h to induce cell injury.

### 2.3. Immunofluorescence

Immunofluorescence staining with *β*III-tubulin antibody was used to observe the morphology changes of the neurite. Briefly, CGNs were washed with HBSS and were fixed with 4% paraformaldehyde for 15 min at 4°C. The cells were then permeabilized with 0.1% Triton X-100 for 5 min. The fixed neurons were incubated in 10% horse serum for 1 h to block nonspecific protein interactions. The primary antibody (rabbit anti-mouse *β*III-tubulin antibody, 1 : 400, Abcam) was added and the cells were incubated at room temperature for 3 h. After washing, the neurons were incubated with the secondary antibody (goat anti-rabbit IgG-FITC, 1 : 100, Invitrogen) for 30 min at room temperature. After washing twice with HBSS, the coverslips were mounted with antiquenching mounting medium and the neurites morphology changes of neurons were visualized using a fluorescence microscope at ×400 magnification.

### 2.4. MTT Assay

The cell viability was assessed using the MTT assay according to conditions described previously [25]. The absorbance at 570 nm was measured using a Wallac Victor3 V microplate reader (PerkinElmer, Netherlands). Cell viability was expressed as a percentage of the MTT reduction of control.

### 2.5. LDH Release Assay

The cytotoxicity inflicted to cells by IAA was also assessed by the LDH release assay. Determination of total and released LDH activity was performed according to the instructions accompanying the cytotoxicity detection kit (Roche). LDH released was normalized to a total LDH release and the results were shown as a percentage of total LDH activity.

### 2.6. FDA/PI Double Staining and Hoechst Staining

Viable neurons were stained with fluorescein formed from FDA, which is deesterified only by living cells. PI can penetrate cell membranes of dead cells to intercalate into double-stranded nucleic acids. Briefly, neurons were washed twice with ice-cold PBS. After incubation with 10 *μ*g/mL of FDA and 5 *μ*g/mL of PI for 15 min, the neurons were examined and photographed using a fluorescence microscope (Nikon Instruments Inc., Melville, NY).

Chromatin condensation was detected by staining the cell nucleus with Hoechst 33342. The cultures were fixed with 4% paraformaldehyde and washed with PBS before staining with Hoechst 33342 for 30 min. Thereafter, cell morphology was observed under a fluorescent microscope (Zeiss, Oberkochen, Germany). The percentage of apoptotic nuclei from five random fields in each well of different treatment groups was quantified and averaged.

### 2.7. Measurement of Mitochondrial Membrane Potential (Δ*ψ*_*m*_)

The dye JC-1 was used as a molecular probe to measure Δ*ψ*_*m*_. The staining procedure was performed according to the manufacturer's instructions with minor modification. Briefly, CGNs were washed with HBSS and stained with 2 *μ*M JC-1 for 10 min. Fluorescence intensity was measured on a microplate reader using 488 nm excitation and 529 nm/590 nm dual emissions. The mitochondrial accumulation of JC-1 is dependent on Δ*ψ*_*m*_ and is reflected by a shift in 529 nm and 590 nm emissions. Mitochondrial membrane depolarization is indicated by a decrease in the ratio of 590 nm to 529 nm emissions.

### 2.8. ROS Measurement

Intracellular ROS were measured using the redox-sensitive fluorescent probe DCFH-DA, which is hydrolyzed to nonfluorescent DCFH by intracellular esterase. DCFH is rapidly oxidized to the fluorescent DCF when reacting with intracellular ROS. CGNs were washed with PBS and were incubated with 20 *μ*M DCF-DA for 1 h. Fluorescence was measured on a microplate reader at an excitation wavelength of 495 nm and an emission wavelength of 515 nm.

### 2.9. Caspase 3 Activity Assay

CGNs were scraped off in HBSS, collected by centrifugation, and lysed at 4°C in cell lysis buffer containing 20 mM EDTA, 20 mM Tris (pH 7.5), and 1% Triton X-100. Lysates were centrifuged at 12000 g at 4°C for 5 min. Caspase 3 assay was performed on 96-well microplates using substrate peptides Ac-DEVD-pNA according to the manufacturer's instruction. The release of p-NA was qualified by determining the absorbance at 405 nm.

### 2.10. Statistical Analysis

All measurements were repeated 3 times. Data were expressed as mean ± SEM and were analyzed using GraphPad Prism V5.0 (GraphPad Software, Inc., San Diego, CA, USA). One-way analysis of variance (ANOVA) and Dunnett's test were used to evaluate statistical differences. The value of statistical significance was set at *P* < 0.05.

## 3. Results

### 3.1. Effect of EDA on IAA-Induced CGNs Death

**Figure 1:**
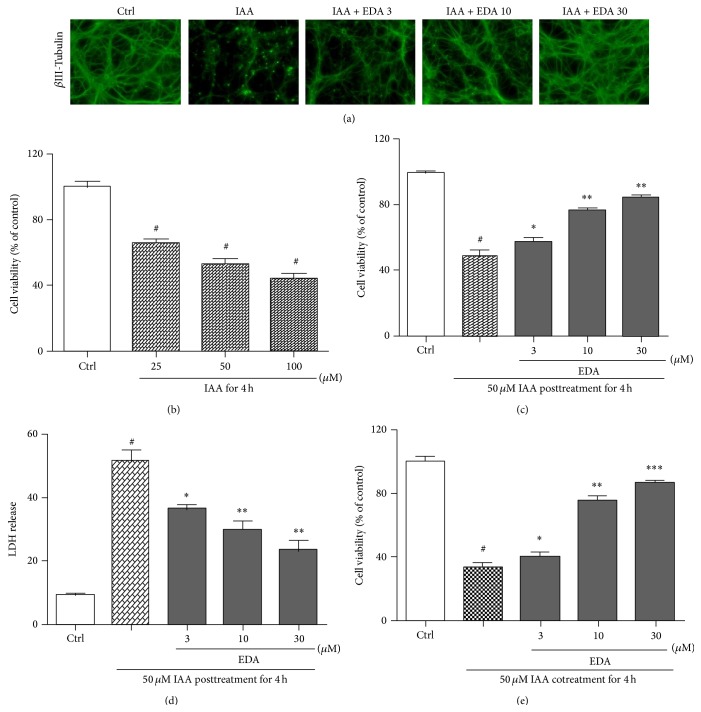
Protective effect of EDA against IAA-induced cells damage in CGNs. (a) Gross morphological change of CGNs cell body and neuritic network was determined by fluorescent immunostaining with antibody against *β*III-tubulin (400x magnification). CGNs were pretreated with or without EDA for 2 h and then incubated with 50 *μ*M IAA for another 4 h. (b) IAA-induced decrease in cell viability by MTT assay. CGNs were exposed to 25, 50, and 100 *μ*M of IAA for 4 h. Cell viability was measured by MTT reduction assay. (c) EDA pretreatment attenuates IAA-induced neuronal loss in a concentration-dependent manner. CGNs were pretreated with or without EDA for 2 h and then incubated with 50 *μ*M IAA for another 4 h. Cell viability was measured by MTT reduction assay. (d) Effect of EDA pretreatment on IAA-induced LDH release. Cells were treated as in (c) and LDH release level was detected by cytotoxicity detection kit. (e) Cotreatment of EDA with IAA inhibits cell viability decrease induced by IAA. CGNs were cotreated with EDA and IAA for 4 h. Cell viability was measured by MTT reduction assay. ^#^ *P* < 0.001 versus control (Ctrl); ^*^ *P* < 0.05; ^**^ *P* < 0.01 and ^***^ *P* < 0.001 versus IAA alone group.

We evaluated the protective effect of EDA on IAA-induced primary neuron death by immunofluorescence-based morphological analysis, MTT reduction and LDH leakage-based cell viability assay, and FDA/PI double staining. As shown in Figure 1(a), IAA clearly destroyed the gross morphology of the CGNs cell body and neuritic network. Pretreatment with EDA notably protected neurons from IAA-induced neurotoxicity (Figure 1(a)). For 4 h treatment, IAA concentration-dependently decreased the cell viability of CGNs. The survival rate reduced approximately by 50% when CGNs were exposed to 50 *μ*M IAA (Figure 1(b)). The cell viability of CGNs pretreated with 3–30 *μ*M EDA significantly increased in a concentration-dependent manner (*P* < 0.05 versus IAA treatment alone, Figure 1(c)) and was up to 85% of control at 30 *μ*M EDA treatment. Pretreatment of EDA also reduced the level of LDH release compared with IAA-treated alone group (*P* < 0.05, Figure 1(d)). Moreover, cotreatment of EDA with IAA also significantly inhibited the decrease of cell viability induced by IAA in a concentration-dependent manner (*P* < 0.05 versus IAA treatment alone, Figure 1(e)).

**Figure 2:**
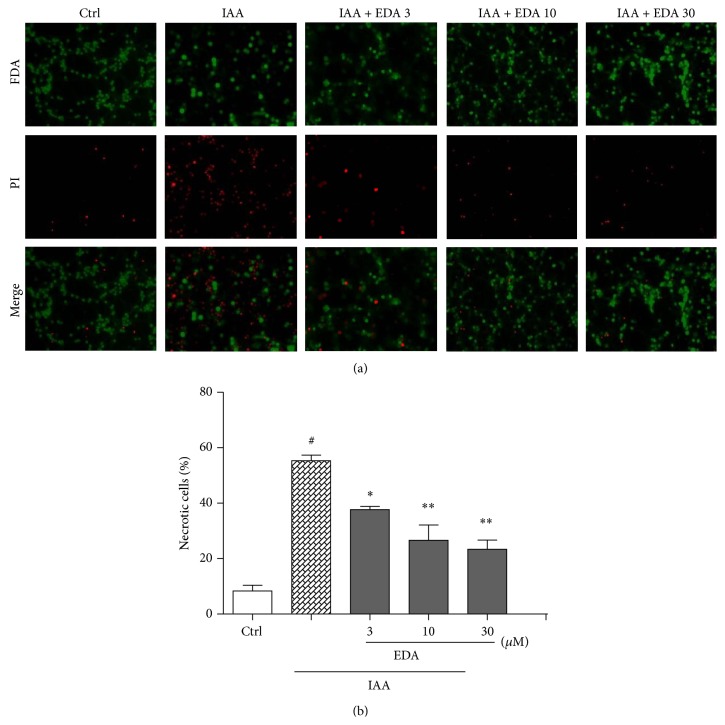
EDA attenuates CGNs neurosis induced by IAA. CGNs were preincubated with or without EDA for 2 h followed by exposure to 50 *μ*M IAA for another 4 h. (a) CGNs were stained with FDA and PI (200x magnification). (b) Quantitative analysis of necrotic cells from three representative photomicrographs was represented as a percentage of the total number of cells counted. ^#^ *P* < 0.001 versus Ctrl; ^*^ *P* < 0.05 and ^**^ *P* < 0.01 versus IAA alone group.

The FDA/PI double staining was performed to further determine whether EDA attenuated cell injury after IAA treatment in CGNs. Simultaneous use of two fluorescent dyes allows a two-color discrimination of the population of live cells from the necrotic-cell population. As shown in Figure 2(a), IAA significantly increased cell necrosis as stained by red fluorescence, accompanying decrease in green fluorescent cells. Consistent with the result of MTT-based cell viability assay, pretreatment of EDA remarkably attenuated CGNs necrosis induced by IAA (Figures 2(a) and 2(b)).

### 3.2. EDA Prevents IAA-Induced CGNs Apoptosis

**Figure 3:**
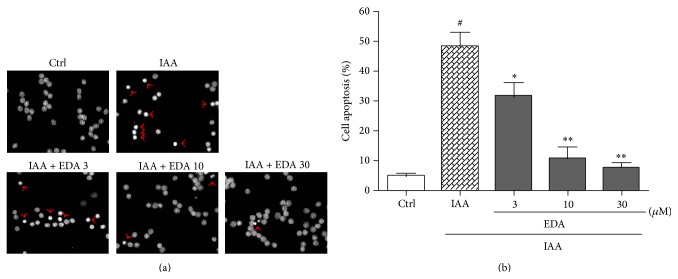
EDA attenuates IAA-induced CGNs apoptosis. CGNs were preincubated with or without EDA for 2 h followed by exposure to 50 *μ*M IAA for another 4 h. (a) Cell apoptosis was assessed by Hoechst 33342 staining and observed by fluorescent microscopy (400x magnification). Apoptotic nuclei with condensed chromatin were indicated by red arrows. (b) The amount of apoptosis nuclei was counted from three representative photomicrographs and was represented as a percentage of the total number of nuclei counted. ^#^ *P* < 0.001 versus Ctrl; ^*^ *P* < 0.05 and ^**^ *P* < 0.01 versus IAA alone group.

Apoptosis is morphologically characterized by cell shrinkage, chromatin condensation. To identify whether EDA reverses IAA-induced CGNs apoptosis, we used Hoechst 33342 staining to evaluate nuclear condensation. As shown in Figure 3(a), normal untreated cells appeared circular or elliptical where no condensation of the nucleus was observable. In contrast, bright condensed dots known as apoptotic bodies (as indicated by arrows) were clearly identified when treated with IAA. Pretreatment of EDA could mitigate IAA-induced apoptosis (Figure 3(a)). In addition, the count of apoptotic nuclei revealed that EDA significantly reduced IAA-induced apoptosis concentration-dependently (Figure 3(b)).

### 3.3. Effects of EDA on IAA-Induced Intracellular ROS Generation

**Figure 4:**
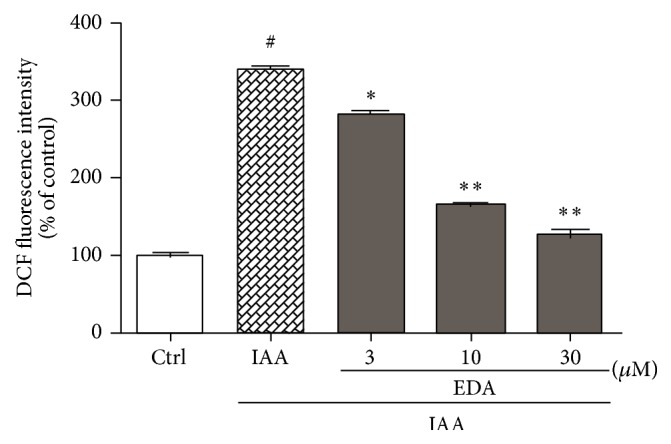
Effect of EDA on IAA-induced ROS production in CGNs. CGNs were preincubated with or without EDA for 2 h followed by exposure to 50 *μ*M IAA for another 4 h. Intracellular ROS generation was evaluated by DCFH-DA probe. ^#^ *P* < 0.001 versus Ctrl; ^*^ *P* < 0.05 and ^**^ *P* < 0.01 versus IAA alone group.

IAA induces cell death accompanied with an increase in ROS production through irreversible inhibition of the glyceraldehyde 3-phosphate dehydrogenase [15–17]. To investigate whether EDA reduces ROS level after IAA treatment in CGNs, intracellular ROS production was detected by DCFH-DA probe. As shown in Figure 4, after exposure to IAA for 4 h, the level of ROS was increased by 3.3-fold in CGNs compared with untreated control. Pretreatment with EDA significantly suppressed this increase in intracellular ROS level. At 30 *μ*M, EDA almost completely inhibited ROS production to a level equal to that of the normal control.

### 3.4. The Effects of EDA on Δ*ψ*_*m*_ in CGNs Induced by IAA

**Figure 5:**
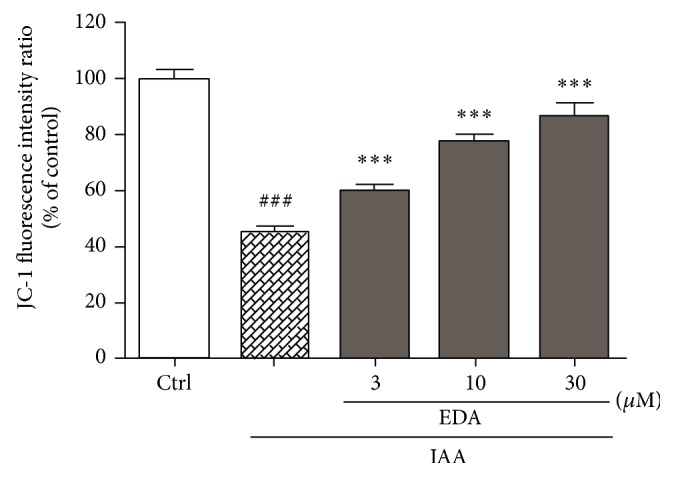
Effect of EDA on IAA-induced Δ*ψ*_*m*_ loss in CGNs. CGNs were preincubated with or without EDA for 2 h followed by exposure to 50 *μ*M IAA for another 4 h. The Δ*ψ*_*m*_ was determined by JC-1. ^###^ *P* < 0.0001 versus control; ^***^ *P* < 0.01 versus IAA alone group.

It has been reported that IAA induces mitochondrial dysfunction [17]. To investigate the protective effect of EDA against IAA-induced mitochondrial damage, Δ*ψ*_*m*_ in CGNs was assessed using JC-1 probe. As shown in Figure 5, IAA treatment resulted in a profound loss of Δ*ψ*_*m*_ in CGNs. When the cells were pretreated with EDA, the Δ*ψ*_*m*_ increased significantly and concentration-dependently compared with that of the IAA alone-treated group.

### 3.5. EDA Attenuates IAA-Induced Caspase 3 Activation in CGNs

**Figure 6:**
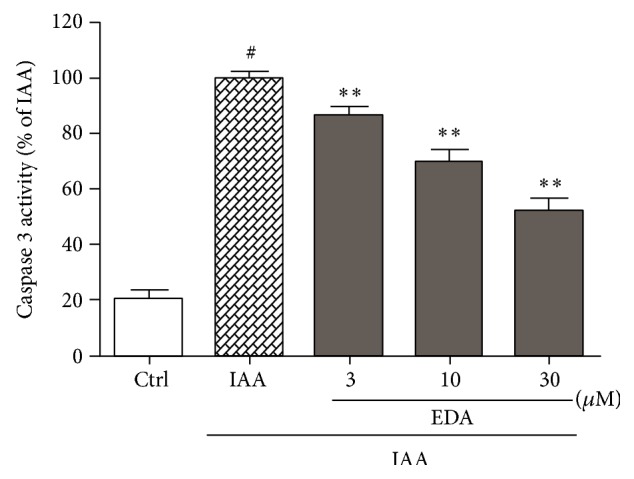
EDA inhibits caspase 3 activation in CGNs. CGNs were preincubated with or without EDA for 2 h followed by exposure to 50 *μ*M IAA for another 4 h. Cells were lysed and caspase 3 activity was measured as described in Section 2.9. ^#^ *P* < 0.001 versus control and ^**^ *P* < 0.01 versus IAA alone group.

Activation of caspase 3 plays a key role in cellular apoptosis.  Figure 6 showed that IAA treatment caused a dramatic increase in caspase 3 activity. When CGNs were preincubated with EDA, the elevated caspase 3 activity induced by IAA was significantly reduced in a concentration-dependent manner.

## 4. Discussion

In the present study, we demonstrated that EDA protected against IAA-induced neurotoxicity in CGNs. Subsequent experiments to explore the mechanisms underlying the neuroprotective effect revealed that EDA significantly decreased neuronal apoptosis and intracellular ROS overproduction, maintained Δ*ψ*_*m*_, and attenuated caspase 3 activity in CGNs.

Cerebral ischemia impairs the normal neurological functions which are triggered by a complex series of biochemical and molecular mechanisms, such as excitotoxicity, oxidative stress, and apoptosis along with histological changes [26]. Cerebral ischemia triggers two general pathways of apoptosis: the intrinsic pathway, originating from mitochondrial release of cytochrome c and associated stimulation of caspase 3, and the extrinsic pathway, originating from the activation of cell surface death receptors, resulting in the stimulation of caspase 8. In addition, cerebral ischemia and reperfusion generate ROS, which causes DNA damage. Many studies have shown that EDA attenuated apoptosis in neuronal cells. Song et al. demonstrated that EDA suppressed the Bad protein overexpression and increased Bcl-2 protein expression, repaired the mitochondrial dysfunction, and maintained ATP level in PC12 cells [27]. Xiong et al. showed that EDA inhibited protein Bax expression and attenuated downregulation of Bcl-XL induced by rotenone [28]. Chen et al. also found that EDA inhibited cobalt chloride-induced apoptosis in PC12 cells via the regulation of Bcl-2 family and reduced caspase 3 activity [4]. Consistent with previous findings, our present study showed that EDA also significantly reduced IAA-induced CGNs apoptosis via suppression of caspase 3 activation and maintaining Δ*ψ*_*m*_. However, different from other studies, which applied oxygen-glucose deprivation, rotenone, and cobalt chloride as causes of cell injury, our present study used IAA to induce cell damage. It has been reported that oxygen-glucose deprivation induced mitochondrial dysfunction and oxidative stress in neurons [29] and stimulated Ca^2+^-dependent glutamate release in cortical slice cultures [30]. Both rotenone and cobalt chloride, as well as IAA, are mitochondrial inhibitors; however, they inhibit mitochondrial function at different sites of the mitochondrial respiratory chain and are inhibitors of mitochondrial respiratory complex I, prolyl hydroxylase, and glyceraldehyde 3-phosphate dehydrogenase, respectively [15, 17, 31, 32].

Free radical production is enhanced in both the ischemic core and penumbra following stroke injury, and this is believed to cause much of the damage seen in these regions [1]. Normally, oxidative stress is being caused by the imbalance between free radical production and degradation. Brain is most susceptible to oxidative stress due to high consumption of oxygen [33]. Natural formation of oxidants during mitochondrial electron transport and autooxidation of some neurotransmitters and in ischemic attacks of events during ischemia can result in oxidant formation and subsequent tissue damage [34]. Normally, free radicals are removed by antioxidant enzymes in living cells. The antioxidant defenses include superoxide dismutase (SOD), glutathione peroxidase (GPX), catalase (CAT), and glutathione (GSH). SOD catalyses the dismutation of the highly reactive superoxide anion (O_2_^∙−^) to O_2_ and H_2_O_2_. CAT reacts with peroxide to form water and molecular oxygen. GPX catalyses the reduction of organic peroxides using GSH and thereby protects cells from oxidative damage. Many studies have shown that protective effect of EDA was associated with the increased SOD activity and CAT, GSH levels in PC12 cells [35, 36]. Our present study indicated that EDA significantly decreased ROS production induced by IAA in CGNs. However, that EDA defenses ROS production induced by IAA in CGNs through direct scavenging ROS or through indirect elevating antioxidative enzymes needs to be further clarified.

## 5. Conclusion

In conclusion, the neuroprotective effect of EDA on IAA-induced neurotoxicity in CGNs was due to its antiapoptotic and antioxidative effects. However, the precise molecular events involved in its antioxidative and antiapoptotic actions need to be further elucidated.
